# Supplementary material for: School Food Environment in Urban Zambia: A Qualitative Analysis of Drivers of Adolescent Food Choices and Their Policy Implications
Source: Int J Environ Res Public Health. 2022 Jun 17;19(12):7460. doi: 10.3390/ijerph19127460 (PMC9224334; doi:10.3390/ijerph19127460)
Supplement: Supplementary file 1 [file ijerph-19-07460-s001.zip › ijerph-1647480-supplementary.pdf]

Supplementary Table S1: Student FGD guide

Food environment dimensions	Question	Probes
Introduction	Which foods are mostly consumed by people your age? Why?	- What kind of food do young people your age consider to be healthy? Why?<br>- What kind of food do young people your age consider to be unhealthy? why?
Availability	Which types of foods and food sources are available in and around your school? Why?	- Which types of foods and food sources would young people want to be available in and around your school and community? Why?<br>- How has the availability of foods been affected during the Covid-19 crisis changed?
Prices	How is the price of different food types that are mainly consumed by young people like you?	- How has the pricing of different food affected during the Covid-19 crisis?
Vendors and product property	What do young people like you consider when deciding on a place to source food from? Why are these factors important?	- What do young people like you consider when deciding what kind of food to buy? Why are these factors important?<br>- How was your decision on what to consider when deciding on a place to source food from impacted by the Covid-19 crisis?
Marketing and regulation	What kind of food marketing strategies have you been exposed to?<br>Accessibility	- What are the challenges that you experience in buying healthy food?<br>- How were these challenges impacted during the Covid-19 crisis?
Affordability	Which food types are you unable to purchase in your current status?	- How do young people like you feel about their ability to buy foods that is healthy?<br>- How was your ability to buy food that is healthy impacted during the Covid-19 crisis?
Convenience	What makes it easy or difficult to eat healthy food?	- What do you feel are some of the challenges people have in preparing or eating healthy foods?

		- How was your ability to prepare health foods impacted during the Covid-19 crisis?
Desirability	Which types of food are young people like you likely to prepare/ consume? Why?	- What are the factors that influence choice of foods young people like you eat?<br>- 23. What would make young people like you to buy or eat healthy foods more often?

Supplementary Table S2: Food environment check list

Vendor type	Present at school (yes/no)	Food types (Mark with an X if food type is sold by vendor)						
		Fast foods[1]	Snacks[2]	*Nshima*	Fruit, fruit juice, fresh salads	Carbonated or sugar sweetened beverages	Milk, yoghurt	Other
Tuck shop								
Katemba								
Grocery								
Supermarket								
Fast food restaurant								
Street vendor								
Other								

[1]Fast foods included fried chicken and chips, sausage and chips, pizza, *sharwama*

[2]Snack included crisps, *jiggies*, cake, scones, biscuits, doughnuts and sweets
